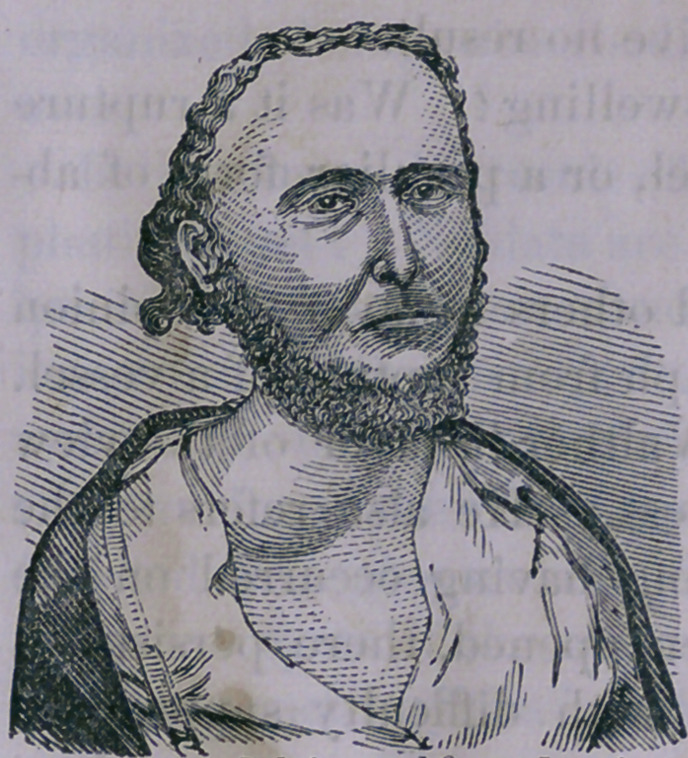# Case of Lymphatic Tumor—Varix of the Lymphatic Trunk

**Published:** 1860-01

**Authors:** Daniel Brainard

**Affiliations:** Professor of Surgery in Rush Medical College, Surgeon of the U. S. Marine Hospital, of the City Hospital, Chicago, etc.


THE CHICAGO

### MEDICAL JOURNAL.

VOL. III. JANUARY, 1860. NO. 1.

### Original Communications.

### ARTICLE I.

### CASE OF LYMPHATIC TUMOR;

### VARIX OF THE LYMPHATIC TRUNK.

BY DANIEL BRAINARD, M. D.,

Professor of Surgery in Rush Medical College, Surgeon of the U. S. Marine Hospital, of the City Hospital, Chicago, etc.

H. W. Johnson, of Chemung, Wisconsin, a physician, aged 48 years, applied to me February 20th, 1847, on account of a tumor situated on the right side of the neck.

This tumor was as large as the "double fists," situated immediately above the clavicle, behind the sterno-mastoid muscle. (*See fig.*) The external jugular vein passed over it. It was perfectly fluctuating, rather soft, free from pain, tenderness or redness. On applying a light, it was seen to be translucent, like a hydrocele. The patient considered himself to be in pretty sound health, although of a sallow complexion.

*History.*—It had originated, without any known cause, some six or eight years previously, and increased very slowly until within a year, during which it has grown more rapidly.

*Treatment.*—I advised, for the purpose of palliation and for testing the character of the tumor, the use of the exploring trochar. It was accordingly punctured with this instrument, and about ten ounces of yellow fluid drawn off, which only about half emptied it. This was of a straw color, resembling exactly in appearance the fluid drawn from a hydrocele, some of which happened to be standing in my room at the time, but on dipping the finger into it, it was felt to have more consistency.

*Microscopic Appearance.*—On examination with the microscope, numerous lymph corpuscles were seen in both the coagulum and the serum. The coagulum was composed of fibrin and corpuscles.

*Chemical Properties.*—Considerable coagulum formed in the lymph on standing. The serous part coagulated very completely by heat. The coagulum presented an arborescent appearance.

Berard states that the solid part of the clot of lymph assumes a purple hue on the contact of carbonic acid, and a bright red on the contact of oxygen.*

This was found to be the case with regard to oxygen when applied to the fibrin, but that used for carbonic acid was unfortunately so much dried as to give no result.

What was the nature of this swelling? Was it a rupture or dilatation of a lymphatic vessel, or a peculiar form of abscess, or a serous cyst?

Chelius quotes Bieul, Rust and others in favor the opinion that it is an extravasation of lymph from rupture of a vessel. On the other hand he quotes Walther in favor of the view that it is an abscess, (lymph abscess.) He also refers to the case of Nasse, in which a swelling having occurred on the upper part of the thigh and been opened, there persisted a discharge of lymph which was with difficulty suppressed. Vol. 1, p. 60.

---

* Cours de Physiologie, vol. 2, p. 778.

Notwithstanding this and other cases of so-called fistular openings of lymphatic vessels, doubts of the nature of such cases may still be entertained.

The case reported by Mr. South, in his notes to Chelius' Surgery, is quite uncertain in its character, for want of a suitable examination of the fluid, the only details given concerning it, being that it was "very similar to synovia."

We may, I think, affirm with safety, that the tumor above described is most unlike an abscess in its history, structure and contents, and in the result of treatment. Nor can the peculiar character of inflammation, when occurring in the lymphatic system, be invoked to account for these peculiarities. The diseases of the lymphatic system, with the exception of varix of the trunks, are well understood. Inflammation, whether occurring in the trunks or ganglions has been studied with great attention since the latter part of the last century. The serous pus formed in cold or scrofulous abscesses, is the well-known result of certain grades of inflammation in the tissues. But nothing approximating to the character of lymph has been found in such abscesses. The conclusion seems, then, irresistible, that this tumor was the result of a rupture or dilatation of a lymphatic vessel.

If I were to adopt a different conclusion, the only alternative would be to suppose that inflammatory lymph may remain indefinitely in the tissues, without degenerating, becoming organized, being absorbed, or suffering any change whatever.

Is the tumor the result of a rupture or dilatation of the lymphatic vessel? No data are furnished by which to resolve this question. On the one hand we might cite the case of aneurism, when there is a rupture of some of the coats of an artery, and on the other, that of varix, where there is dilatation of the covering of the vein. It seems, however, that the probabilities and analogies are altogether in favor of dilatation.

Serous cysts of the neck are not by any means rare, and their treatment by the injection of iodine has been resorted to with success. The reactions of the fibrinous parts of the contents of this tumor appear to me sufficiently conclusive as to its character. If it was a lymphatic tumor, the treatment was, so far as I am aware, new.

*April* 21, 1857 —Patient returned. Tumor nearly as large as before.

Tapped with a common hydrocele trochar, and drew off 12 ounces of fluid, which completely emptied it. Injected a small quantity of solution composed of Iodine one scruple, Iodide of Potassium one drachm, to one ounce of water. A small quantity remained. This produced an acute pain, shooting down into the shoulder, and up into the neck.

The fluid in this case was more turbid than at the former puncture; coagulated, but the coagulum did not separate as before, but resembled jelly.

The following note from the patient, written eighteen months after the operation, will sufficiently show the result:

—

HOWARD, McHenry Co., Ill., Dec. 27, 1858.

DEAR SIR:

Your kind letter of Dec. 3d has just arrived, inquiring as to the result of your injection of Iodine into the sack of a tumor on the shoulder. I am very happy to inform you it succeeded admirably. In about sixty days, it contracted the sac down to the size of a penny; in from three to four months the last vestige of it disappeared, without further treatment, and it is at this time perfectly well and smooth as the other side. It remained somewhat sore and numb for five or six months from the time of operation.

And in conclusion, allow me to express my heartfelt satisfaction for your kindness, as well as the correctness of your diagnosis, and the ability and care with which you affected the cure; for it, as to its results, had become the source of a good deal of annoyance to me. My general health is as good as it ever was. One thing I forgot to mention to you at the time, not thinking it connected with the tumor, viz., bloody urine for two years previous to the operation, that ceased altogether as the tumor disappeared. Could it have been the result of, or in any way connected with, the growth of the tumor?

Any further information that you may require, will be cordially responded to. Three months from the time you operated, I wrote a full account of it, but I suppose you never received it, from the fact I never heard from you till now. Drop me a line as soon as possible, so I shall know you have received this.

H. W. JOHNSON.

D. Brainard, M. D.

## Figures and Tables

**Figure f1:**